# Supplementary material for: Unveiling the Bioactive Potential of the Invasive Jellyfish Phyllorhiza punctata Through Integrative Transcriptomic and Proteomic Analyses
Source: Biomolecules. 2025 Aug 4;15(8):1121. doi: 10.3390/biom15081121 (PMC12383608; doi:10.3390/biom15081121)
Supplement: Supplementary file 1 [file biomolecules-15-01121-s001.zip › Suplementary Material/FileS3/PEPFOLD4/XP_065051094_793/clusters.html]

 PEP-FOLD clusters


Download archive of all models  here   

|  |  |  |
| --- | --- | --- |
| # | file | sOPEP |
| # cluster 1 - Size: 3 |  |  |
|  | XP\_065051094\_793-model1 | -16.8329 |
|  | XP\_065051094\_793-model1.1 | -13.8697 |
|  | XP\_065051094\_793-model1.2 | -10.3784 |
| # cluster 2 - Size: 2 |  |  |
|  | XP\_065051094\_793-model2 | -16.798 |
|  | XP\_065051094\_793-model2.1 | -16.3245 |
| # cluster 3 - Size: 14 |  |  |
|  | XP\_065051094\_793-model3 | -15.5619 |
|  | XP\_065051094\_793-model3.1 | -15.2691 |
|  | XP\_065051094\_793-model3.2 | -14.3258 |
|  | XP\_065051094\_793-model3.3 | -14.0708 |
|  | XP\_065051094\_793-model3.4 | -12.5976 |
|  | XP\_065051094\_793-model3.5 | -12.4639 |
|  | XP\_065051094\_793-model3.6 | -12.3857 |
|  | XP\_065051094\_793-model3.7 | -12.3404 |
|  | XP\_065051094\_793-model3.8 | -11.0161 |
|  | XP\_065051094\_793-model3.9 | -10.9749 |
|  | XP\_065051094\_793-model3.10 | -10.554 |
|  | XP\_065051094\_793-model3.11 | -9.71679 |
|  | XP\_065051094\_793-model3.12 | -9.57305 |
|  | XP\_065051094\_793-model3.13 | -8.87804 |
| # cluster 4 - Size: 2 |  |  |
|  | XP\_065051094\_793-model4 | -15.5358 |
|  | XP\_065051094\_793-model4.1 | -15.2586 |
| # cluster 5 - Size: 2 |  |  |
|  | XP\_065051094\_793-model5 | -14.6666 |
|  | XP\_065051094\_793-model5.1 | -13.6185 |
| # cluster 6 - Size: 2 |  |  |
|  | XP\_065051094\_793-model6 | -14.5866 |
|  | XP\_065051094\_793-model6.1 | -14.5384 |
| # cluster 7 - Size: 1 |  |  |
|  | XP\_065051094\_793-model7 | -14.5624 |
| # cluster 8 - Size: 2 |  |  |
|  | XP\_065051094\_793-model8 | -14.5228 |
|  | XP\_065051094\_793-model8.1 | -13.4058 |
| # cluster 9 - Size: 2 |  |  |
|  | XP\_065051094\_793-model9 | -14.0536 |
|  | XP\_065051094\_793-model9.1 | -9.21431 |
| # cluster 10 - Size: 1 |  |  |
|  | XP\_065051094\_793-model10 | -13.9672 |
| # cluster 11 - Size: 1 |  |  |
|  | XP\_065051094\_793-model11 | -13.5984 |
| # cluster 12 - Size: 11 |  |  |
|  | XP\_065051094\_793-model12 | -13.4799 |
|  | XP\_065051094\_793-model12.1 | -13.4188 |
|  | XP\_065051094\_793-model12.2 | -13.3748 |
|  | XP\_065051094\_793-model12.3 | -12.1885 |
|  | XP\_065051094\_793-model12.4 | -11.3839 |
|  | XP\_065051094\_793-model12.5 | -10.8178 |
|  | XP\_065051094\_793-model12.6 | -10.369 |
|  | XP\_065051094\_793-model12.7 | -10.2197 |
|  | XP\_065051094\_793-model12.8 | -10.035 |
|  | XP\_065051094\_793-model12.9 | -9.91842 |
|  | XP\_065051094\_793-model12.10 | -9.87024 |
| # cluster 13 - Size: 7 |  |  |
|  | XP\_065051094\_793-model13 | -13.4144 |
|  | XP\_065051094\_793-model13.1 | -12.9356 |
|  | XP\_065051094\_793-model13.2 | -12.4056 |
|  | XP\_065051094\_793-model13.3 | -12.1266 |
|  | XP\_065051094\_793-model13.4 | -10.7029 |
|  | XP\_065051094\_793-model13.5 | -10.6692 |
|  | XP\_065051094\_793-model13.6 | -10.3991 |
| # cluster 14 - Size: 3 |  |  |
|  | XP\_065051094\_793-model14 | -13.3941 |
|  | XP\_065051094\_793-model14.1 | -11.7176 |
|  | XP\_065051094\_793-model14.2 | -11.3051 |
| # cluster 15 - Size: 3 |  |  |
|  | XP\_065051094\_793-model15 | -13.3912 |
|  | XP\_065051094\_793-model15.1 | -12.1369 |
|  | XP\_065051094\_793-model15.2 | -9.66048 |
| # cluster 16 - Size: 1 |  |  |
|  | XP\_065051094\_793-model16 | -12.9278 |
| # cluster 17 - Size: 1 |  |  |
|  | XP\_065051094\_793-model17 | -12.9082 |
| # cluster 18 - Size: 2 |  |  |
|  | XP\_065051094\_793-model18 | -12.7433 |
|  | XP\_065051094\_793-model18.1 | -10.3544 |
| # cluster 19 - Size: 1 |  |  |
|  | XP\_065051094\_793-model19 | -12.7233 |
| # cluster 20 - Size: 4 |  |  |
|  | XP\_065051094\_793-model20 | -12.5786 |
|  | XP\_065051094\_793-model20.1 | -11.8648 |
|  | XP\_065051094\_793-model20.2 | -11.1576 |
|  | XP\_065051094\_793-model20.3 | -10.447 |
| # cluster 21 - Size: 3 |  |  |
|  | XP\_065051094\_793-model21 | -12.5326 |
|  | XP\_065051094\_793-model21.1 | -11.1226 |
|  | XP\_065051094\_793-model21.2 | -9.75343 |
| # cluster 22 - Size: 1 |  |  |
|  | XP\_065051094\_793-model22 | -12.5096 |
| # cluster 23 - Size: 2 |  |  |
|  | XP\_065051094\_793-model23 | -12.4687 |
|  | XP\_065051094\_793-model23.1 | -10.1332 |
| # cluster 24 - Size: 2 |  |  |
|  | XP\_065051094\_793-model24 | -12.4047 |
|  | XP\_065051094\_793-model24.1 | -9.66321 |
| # cluster 25 - Size: 2 |  |  |
|  | XP\_065051094\_793-model25 | -12.2454 |
|  | XP\_065051094\_793-model25.1 | -9.18642 |
| # cluster 26 - Size: 1 |  |  |
|  | XP\_065051094\_793-model26 | -12.0537 |
| # cluster 27 - Size: 3 |  |  |
|  | XP\_065051094\_793-model27 | -12.0291 |
|  | XP\_065051094\_793-model27.1 | -9.78309 |
|  | XP\_065051094\_793-model27.2 | -9.34829 |
| # cluster 28 - Size: 5 |  |  |
|  | XP\_065051094\_793-model28 | -11.193 |
|  | XP\_065051094\_793-model28.1 | -10.7739 |
|  | XP\_065051094\_793-model28.2 | -10.7701 |
|  | XP\_065051094\_793-model28.3 | -10.3708 |
|  | XP\_065051094\_793-model28.4 | -10.3074 |
| # cluster 29 - Size: 1 |  |  |
|  | XP\_065051094\_793-model29 | -11.0524 |
| # cluster 30 - Size: 1 |  |  |
|  | XP\_065051094\_793-model30 | -10.7897 |
| # cluster 31 - Size: 1 |  |  |
|  | XP\_065051094\_793-model31 | -10.7793 |
| # cluster 32 - Size: 1 |  |  |
|  | XP\_065051094\_793-model32 | -10.5624 |
| # cluster 33 - Size: 1 |  |  |
|  | XP\_065051094\_793-model33 | -10.4112 |
| # cluster 34 - Size: 1 |  |  |
|  | XP\_065051094\_793-model34 | -10.3751 |
| # cluster 35 - Size: 1 |  |  |
|  | XP\_065051094\_793-model35 | -10.1966 |
| # cluster 36 - Size: 1 |  |  |
|  | XP\_065051094\_793-model36 | -9.77873 |
| # cluster 37 - Size: 1 |  |  |
|  | XP\_065051094\_793-model37 | -9.77599 |
| # cluster 38 - Size: 1 |  |  |
|  | XP\_065051094\_793-model38 | -9.3707 |
| # cluster 39 - Size: 1 |  |  |
|  | XP\_065051094\_793-model39 | -9.28646 |
| # cluster 40 - Size: 1 |  |  |
|  | XP\_065051094\_793-model40 | -9.17217 |
| # cluster 41 - Size: 1 |  |  |
|  | XP\_065051094\_793-model41 | -9.10352 |
| # cluster 42 - Size: 2 |  |  |
|  | XP\_065051094\_793-model42 | -9.09494 |
|  | XP\_065051094\_793-model42.1 | -8.75563 |
| # cluster 43 - Size: 1 |  |  |
|  | XP\_065051094\_793-model43 | -9.04901 |
|  |  |  |
